# ASO Author Reflections: Enhancing Palliative Care Delivery: The Potential of Multi-Disciplinary Intervention Teams for Surgical Oncology Patients

**DOI:** 10.1245/s10434-023-14198-5

**Published:** 2023-09-05

**Authors:** Darryl W. K. Juan, Joel J. Y. Soon, Jolene S. M. Wong

**Affiliations:** 1 Department of Sarcoma, Peritoneal and Rare Tumours (SPRinT), Division of Surgery and Surgical Oncology, National Cancer Centre Singapore, Singapore, Singapore; 2 Department of Sarcoma, Peritoneal and Rare Tumours (SPRinT), Division of Surgery and Surgical Oncology, Singapore General Hospital, Singapore, Singapore; 3 Division of Surgery and Surgical Oncology, Singapore General Hospital, Singapore, Singapore; 4 Duke-NUS Medical School, SingHealth Duke-NUS Oncology Academic Clinical Program, Singapore, Singapore

## Past

Palliative surgical oncology patients face a wide range of challenges, such as complex decision-making processes and unique end-of-life (EOL) concerns.^1^ Although the involvement of a multidisciplinary team comprising specialized palliative care physicians and other healthcare providers has been shown to improve health-related quality of life (HRQoL) for medical patients,^2^ its utility and feasibility in caring for palliative surgical oncology patients is still unknown.

## Present

We established a multidisciplinary palliative surgical intervention (MD-PALS) team comprising healthcare providers of various subspecialties involved in the care of these patients. We then performed a single-centre prospective cohort study recruiting advanced cancer patients who received palliative interventions, comparing the outcomes of those cared for under the newly established MD-PALS team and those who received usual care.^3^ Rather than comparing traditional surgical outcome measures that often fall short of measuring what is meaningful to patients during EOL,^4^ we assessed the quality of Goals of Care (GOC) discussions, which are associated with improved patient HRQoL and enhanced goal-concordant care.^5^ We found that MD-PALS led to a significantly higher quality of GOC discussion composite scores compared with those who received usual care (2.61 vs. 1.34; *p* < 0.001), representing better GOC discussions with patients. This could be due to the primary surgical oncologist informed by MD-PALS members being able to better identify key EOL issues surrounding the patient and hence better equipped to conduct a quality GOC discussion during a surgical admission.

## Future

Our study highlights the positive impact of multidisciplinary specialist providers’ involvement in the care of palliative surgical oncology patients, leading to enhanced GOC discussions. MD-PALS teams hold the potential to become the cornerstone of a ‘Community of Practice’, fostering interdisciplinary collaboration and seamless communication between team members, patients, and their families, ensuring the comprehensive and holistic management of the multifaceted challenges faced by these patients.^6^ Future research should delve deeper into investigating and validating the other potential benefits of MD-PALS teams. Robust and rigorous studies will be essential in refining team structures and processes, ensuring the model is optimized to cater to the unique needs of palliative surgical oncology patients.
